# Evaluation of the impact of rifampicin on the plasma concentration of linezolid in tuberculosis co-infected patients

**DOI:** 10.3389/fphar.2023.1260535

**Published:** 2023-11-07

**Authors:** Pan Yan, Qun-Zhi Shi, Yi-Xing Hu, Ying Zeng, Hong Lu

**Affiliations:** Department of Pharmacy, The Affiliated Changsha Central Hospital, Hengyang Medical School, University of South China, Changsha, China

**Keywords:** linezolid, rifampicin, interaction, plasma concentration, tuberculosis

## Abstract

Linezolid combined with rifampicin has shown excellent clinical outcomes against infection by multi-resistant Gram-positive bacteria. However, several studies have indicated that rifampicin reduces the plasma concentration of linezolid in patients with severe infection. Linezolid has been recommended for the treatment of patients with multidrug-resistant or extensively drug-resistant tuberculosis. However, studies on the interaction between linezolid and rifampicin in patients suffering from tuberculosis with infection are lacking. We evaluated the interaction between linezolid and rifampicin based on therapeutic drug monitoring (TDM). A retrospective analysis was undertaken for patients with tuberculosis and infection who were treated with linezolid and undergoing TDM. Patients were divided into the linezolid group and linezolid + rifampicin group. Data on demographic characteristics, disease, duration of linezolid therapy, and the plasma concentration of linezolid were used for statistical analyses. Eighty-eight patients with tuberculosis and infection were assessed. Values for the peak (C_max_) and trough (C_min_) concentrations of linezolid in plasma were available for 42 and 46 cases, respectively. Patients in the linezolid group had a significantly higher C_max_ [15.76 (8.07–26.06) vs. 13.18 (7.48–23.64) mg/L, *p* = 0.048] and C_min_ [8.38 (3.06–16.53) vs. 4.27 (0.45–10.47), *p* = 0.005] than those in the linezolid + rifampicin group. The plasma concentration of linezolid increased obviously in two patients after rifampicin discontinuation. However, the total efficiency and prevalence of hematologic adverse reactions were not significantly different in the linezolid group and linezolid + rifampin group. The plasma concentration of linezolid decreased upon combination with rifampicin, suggesting that TDM may aid avoidance of subtherapeutic levels of linezolid upon co-treatment with rifampicin.

## Introduction

Linezolid was the first antibacterial drug of the oxazolidinone class to be used clinically. It can inhibit the synthesis of bacterial proteins. Linezolid has a unique site and mode of action. Hence, cross-resistance with other inhibitors of protein synthesis and anti-tuberculosis drugs is unlikely, and it does not induce resistance readily *in vitro* (Singh et al., 2019). *In vitro* research suggests that linezolid has a strong effect against *M. tuberculosis*, and a minimum inhibitory concentration (MIC) of 0.125–1.0 mg/L has been documented. It has equal activity against sensitive and resistant strains of *Mycobacterium tuberculosis*, and an effect against fast-growing bacteria and quiescent bacteria. Therefore, linezolid has become the main drug for long-term treatment for drug-resistant tuberculosis (TB) (Tuberculosis and Association, 2022). The oxidative metabolism of linezolid is non-enzymatic, and does not involve the cytochrome P450 (CYP) enzymes in hepatic microsomes. Clinical research has shown that ∼65% of linezolid is cleared by non-renal mechanisms, and ∼30% is excreted in urine (Slatter et al., 2001).

Rifampicin is the first-line drug used to counteract tuberculosis. It is a strong inducer of CYP and the P-glycoprotein transport system. Rifampicin has enzyme-inducing effects on CYP3A, CYP1A2, CYP2C, and CYP2D6, which leads to significant interactions with several drug types (Burman et al., 2001; Finch et al., 2002). A combination of vancomycin and rifampicin is usually recommended for the treatment of methicillin-resistant *Staphylococcus aureus* infections in endocarditis, osteomyelitis, and septic arthritis, whereas linezolid may be used if vancomycin is resistant (or ineffective) or if sequential therapy is required (Liu et al., 2011).

There have been several reports of interactions between linezolid with rifampicin, which have indicated that rifampicin reduces the plasma concentration of linezolid and poses a risk of therapeutic failure (Egle et al., 2005; Gebhart et al., 2007; Hoyo et al., 2012). Furthermore, the medication package insert for linezolid glucose injection (Zyvox™; Pfizer Pharmaceuticals, New York, NY, United Staes) states that patients receiving rifampicin with linezolid (p.o.) results in a 21% and 32% reduction in the peak serum concentration (C_max_) and area under the drug concentration–time curve (AUC), respectively, for linezolid, but the clinical importance of this interaction is not known. The mechanism by which linezolid and rifampicin interact is not known. Moreover, reports on co-administration of linezolid and rifampicin in TB patients co-infected with other bacterial pathogens are lacking.

We undertook a retrospective analysis to reveal the effect of co-administration of rifampicin on the plasma concentration of linezolid in patients suffering from tuberculosis and infection. We wished to evaluate the efficacy and safety of linezolid and rifampicin if administered alone or in combination.

## Materials and methods

### Setting

This observational retrospective study was undertaken at Affiliated Changsha Central Hospital (ACCH) within the University of South China (Hengyang, China). ACCH, a grade-IIIA hospital, is a key medical research center for tuberculosis in Hunan province. The Clinical Pharmacology Department answers all requests for therapeutic drug monitoring (TDM) for ACCH and its healthcare area.

### Inclusion and exclusion criteria

The inclusion criteria were patients: 1) aged ≥18 years; 2) receiving continuous linezolid injection with a standard administration [(600 mg every 12 h (Q12h)] for treatment >3 days; 3) for whom TDM results for linezolid at steady state were available.

The exclusion criteria were patients: 1) aged <18 years; 2) who were pregnant or lactating; 3) for whom the plasma concentration of linezolid did not reach a steady state or was below the limit of detection; 4) who were administered other drugs that strongly induced hepatic enzymes (e.g., carbamazepine, phenytoin sodium, phenobarbital) simultaneously; 5) undergoing blood purification or other forms of kidney-replacement therapy.

### Patients

Cases from the Tuberculosis Diagnosis and Treatment Center of ACCH from January 2020 to June 2023 treated with linezolid injection (600 mg, Q12h) and for whom the plasma concentration of linezolid during hospitalization were monitored.

Patients were divided into two groups (linezolid and linezolid + rifampicin) according to whether the medication plan during hospitalization contained rifampicin.

### Determination of drug concentration in plasma

When linezolid had reached a steady-state concentration (≥3 days), blood was collected 30 min before and 30 min after the next intravenous drip to monitor the trough concentration (C_min_) and C_max_, respectively. Plasma was obtained from blood by centrifugation (3,000 × *g* for 10 min at 4°C). Plasma was processed by protein precipitation. Then, high-performance liquid chromatography using a photodiode array (HPLC-PDA) was undertaken. Chromatographic separation was carried out on a Diamonsil C18 column (4.6 × 20 mm, 5 μm). The column oven was set at 30°C. The mobile phase consisted of methanol: water (40:60). The detection wavelength was 253 nm.

The analytical method met the requirements for determination of biological samples, with absolute recovery >85% and a linear range of 0.31–40.55 mg/L (R^2^ = 0.9997). The intra-day precision and inter-day precision of low, medium, and high concentrations were <5%. Biological samples were stable within 24 h at room temperature, at two freeze–thaw cycles, and after freezing at −80°C for 3 months. The C_min_ range of linezolid is 2–8 mg/L, whereas the C_max_ range is 12–26 mg/L (Beijin Chest Hospital and Antituberculosis, 2021; Lin et al., 2022).

### Data collection

The electronic medical record system (EMRS) of ACCH was used to retrieve and collect patient information. The data collected were organized as: 1) demographics (age, sex, bodyweight); 2) reason and time for administration of linezolid injection; 3) pathogen information; 4) detection time and results for the plasma concentration of linezolid; 5) laboratory data for liver function (albumin, total bilirubin), renal function (creatinine), and blood data before and after medication administration (white blood cell (WBC) count, percent neutrophils, hemoglobin, platelet count).

### Clinical outcome

After anti-tuberculosis and anti-infection treatment with linezolid, if symptoms disappeared or improved significantly, pathogenic bacteria were cleared, or imaging suggested improvement, then this scenario was classified as “clinically effective”; if not, then the classification was “clinically ineffective.”

### Safety and tolerability of linezolid

Hematological adverse drug reactions (ADRs) and peripheral neuropathy were monitored. The physician presumed that the ADR was due to linezolid, then reduced the dose or stopped linezolid.

A hematological ADRs was defined as: 1) a reduction in the platelet count and/or hemoglobin level >30% in comparison with those at baseline; 2) a normal or high WBC count 3 days before or on the day of dosing but a WBC count below the lower limit of normal (3.5 × 10^9^/L) after dosing (Takahashi et al., 2011).

Peripheral neuropathy was defined as follows: 1) patients were suspected to have developed neuropathy if the EMRS demonstrated nerve dysfunctions (pain, sensory loss, or numbness); 2) neurological symptoms reduced or disappeared upon withdrawal of linezolid.

### Statistical analyses

Continuous variables are presented as the mean ± SD or median according to the normality (Kolmogorov–Smirnov) test. To estimate differences between variables, the chi-squared test and Student´s *t*-test were used for categorical variables and continuous parametric variables, respectively. Statistical analyses were undertaken using Prism 8 (GraphPad, San Diego, United States). *p* < 0.05 was considered significant.

### Ethical statement

The study protocol was approved (2023-003) by the research ethics committee of the University of South China. The requirement for informed consent was waived because we collected data retrospectively.

## Results

### Patient characteristics

Eighty-eight tuberculosis patients with infection for whom the plasma concentration of linezolid were monitored. 42 patients were in the linezolid group and 46 cases in the linezolid + rifampicin group, in which the C_max_ and C_min_ of linezolid were obtained for 57 and 31 cases, respectively. The characteristics of patients are displayed in Tables 1, 2. Except for the platelet level at baseline before dosing, there were no significant differences in the basic characteristics of patients and reasons for using linezolid. The main reason for linezolid administration was to counteract tuberculous and to counteract tuberculosis and infection. Microbiological isolates were indentified in 30.95% and 15.22% of patients in the linezolid group and linezolid + rifampicin group, respectively, and *Enterococcus faecium* and *M. tuberculosis* were the most prevalent pathogens in both groups.

**TABLE 1:** Characteristics of C_max_ of linezolid in the two groups.

	Linezolid group	Linezolid + rifampicin group	*p*-value
	(*n* = 23)	(*n* = 34)	
Age, years	57.70 ± 16.92	49.24 ± 20.55	0.108
Sex (Male/Female)	16/7	21/13	0.985
Albumin, g/L	29.65 ± 6.94	31.50 ± 5.33	0.261
TBIL, μmol/L	13.30 ± 12.58	10.41 ± 7.80	0.288
Creatinine, μmol/L	72.48 ± 51.21	60.91 ± 34.37	0.311
eGFR, mL/min/1.73m^2^	103.35 ± 29.82	109.31 ± 27.96	0.445
White blood cell (10^9^/L)^a^	9.20 ± 5.41	9.68 ± 5.11	0.736
Neutrophil percent (%)^a^	80.03 ± 13.67	80.46 ± 10.20	0.890
Hemoglobin (g/L)^a^	95.17 ± 22.59	102.74 ± 19.66	0.185
Blood platelet (10^9^/L)^a^	250.48 ± 91.70	284.12 ± 130.63	0.290
Main Reasons for linezolid, n (%)			
Anti-infection + Antituberculous	14 (60.87)	11 (32.36)	0.104
Antituberculous	6 (26.09)	20 (58.82)	0.052
Anti-infection	3 (13.04)	3 (8.82)	0.878
Pathogenic bacteria, n (%)			
*Mycobacterium tuberculosis*	4 (17.39)	2 (5.88)	0.933
*Enterococcus faecium*	1 (4.35)	4 (11.76)	0.667
*Enterococcus faecalis*	1 (4.35)	0 (0)	NA
Undefined diagnosis	17 (73.91)	28 (82.36)	0.745

^a^ On the day of medication (or 3 days before medication); TBIL, total bilirubin; eGFR, estimated glomerular filtration rate.

**TABLE 2:** Characteristics of C_min_ of linezolid in the two groups.

	Linezolid group	Linezolid + rifampicin group	*p*-value
	(*n* = 19)	(*n* = 12)	
Age, years	62.21 ± 21.52	53.08 ± 24.49	0.284
Sex (Male/Female)	15/4	9/3	0.968
Albumin, g/L	26.26 ± 5.44	29.83 ± 6.53	0.111
TBIL, μmol/L	10.56 ± 7.20	12.54 ± 11.33	0.557
Creatinine, μmol/L	70.00 ± 46.33	59.83 ± 37.96	0.530
eGFR, mL/min/1.73m^2^	99.62 ± 27.94	107.99 ± 34.87	0.466
White blood cell (10^9^/L)^#^	12.24 ± 7.66	13.60 ± 7.26	0.627
Neutrophil percent (%)^#^	83.81 ± 10.10	83.86 ± 9.03	0.989
Hemoglobin (g/L)^#^	91.21 ± 16.64	102.00 ± 22.14	0.133
Blood platelet (10^9^/L)^#^	226.47 ± 71.88	372.83 ± 199.61	0.007^**^
Main Reasons for linezolid, n (%)			
Anti-infection + antituberculous	10 (52.63)	7 (58.33)	0.953
Antituberculous	6 (31.58)	5 (41.67)	0.849
Anti-infection	3 (15.79)	0	NA
Pathogenic bacteria, n (%)			
*Enterococcus faecium*	4 (21.05)	1 (8.33)	0.713
*Mycobacterium tuberculosis*	1 (5.26)	0 (0)	NA
*Mycobacterium abscess*	1 (5.26)	0 (0)	NA
*Staphylococcus haemolyticus*	1 (5.26)	0 (0)	NA
Undefined diagnosis	12 (63.16)	11 (91.67)	0.210

* *p* < 0.05; **, *p* < 0.01 #, On the day of medication (or 3 days before medication); TB, total bilirubin; GFR, glomerular filtration rate.

### Distribution of the plasma concentration of linezolid

The times when the C_max_ and C_min_ were monitored in the linezolid and linezolid + rifampicin groups were different, and the results are shown in Table 3. The monitoring time (median) of C_max_ in linezolid and linezolid + rifampicin groups was 6 days and 10 days respectively, whereas the C_min_ was 5 days and 12.5 days, respectively. The median values for the monitoring time of C_max_ and C_min_ were significantly longer in the linezolid + rifampicin group than in the linezolid group, but the mean values of C_max_ and C_min_ in the linezolid group were significantly higher than those in the linezolid + rifampicin group [(15.76 ± 5.77) vs. (13.18 ± 3.88) mg/L and (8.38 ± 4.04) vs. (4.27 ± 3.00) mg/L]. Furthermore, there was a significant difference in the distribution of C_min_ values between the two groups. In the linezolid group, 47.37% (9/19) of patients had a higher C_min_, whereas no patients had a lower C_min_. However, 16.67% (2/12) of patients in the linezolid + rifampicin group had a higher C_min_, whereas 25.00% (3/12) patients had a lower C_min_. These results implied that the plasma concentration of linezolid in the linezolid + rifampicin group was lower than those in the linezolid group.

**TABLE 3:** Distribution of the plasma concentration of linezolid.

	C_max_ group		*p-*value	C_min_ group		*p-*value
	Linezolid group (n = 23)	Linezolid + rifampicin group (n = 34)		Linezolid group (n = 19)	Linezolid + rifampicin group (n = 12)	
Linezolid plasma concentrations (mg/L)	15.76 ± 5.77	13.18 ± 3.88	0.048^*^	8.38 ± 4.04	4.27 ± 3.00	0.005^**^
Number of TDM (days) median (IQR)	6 (3–28)	10 (3–48)	0.057	5 (3–10)	12.5 (4–47)	0.001^**^
Length of treatment (days), median (IQR)	11 (4–67)	17 (5–62)	0.307	9 (5–31)	29 (8–62)	0.001^**^
Plasma concentration distribution, n (%)			0.624			0.034^*^
High concentration	1 (4.35)	0 (0)		9 (47.37)	2 (16.67)	
Normal concentration	12 (52.17)	23 (67.65)		10 (52.63)	7 (58.33)	
Low concentration	10 (43.48)	11 (32.35)		0 (0)	3 (25.00)	
Co-treatment, n (%)						
Omeprazole	2 (8.70)	15 (44.12)	0.084	5 (26.32)	6 (50.00)	0.406
Amlodipine	2 (8.70)	6 (17.65)	0.453	3 (15.79)	3 (25.00)	0.818

* *p* < 0.05; **, *p* < 0.01.

### Efficacy and safety of linezolid

With respect to C_max_, the proportion of hematological ADRs was 43.48% (10/23) and 44.12% (15/34) in the linezolid group and linezolid + rifampin group, respectively, and three patients in both groups experienced reductions in the platelet count and WBC count. With regard to C_min_, the proportion of hematological ADRs was 47.37% (9/19) and 66.67% (8/12) in the linezolid group and linezolid + rifampin group, respectively, whereas two patients in the linezolid group and three patients in linezolid + rifampicin group experienced two reductions in three blood indicators. Meanwhile, we found the same clinical efficacy [73.81% (31/42)] in the linezolid group compared with that in the linezolid + rifampicin group [73.91% (34/46)]. In general, there was no significant difference in the prevalence of clinical efficacy or ADRs between the two groups (Table 4).

**TABLE 4:** Evalution of efficacy and adverse drug reactions.

	C_max_ group		*p-*value	C_min_ group		*p-*value
	Linezolid group (n = 23)	Linezolid + rifampicin group (n = 34)		Linezolid group (n = 19)	Linezolid + rifampicin group (n = 12)	
Clinical improvement, n (%)	15 (65.22)	26 (76.47)	0.650	16 (84.21)	8 (66.67)	0.523
ADRs, n (%)	10 (43.48)	15 (44.12)	0.999	9 (47.37)	8 (66.67)	0.575
Thrombocytopenia, n (%)	9 (39.13)	15 (44.12)	0.932	7 (36.84)	7 (58.33)	0.504
Anaemia, n (%)	1 (4.35)	1 (2.94)	0.900	2 (10.53)	2 (16.67)	0.845
White blood cell decline, n (%)	3 (13.04)	3 (8.82)	0.878	2 (10.53)	2 (16.67)	0.884
Peripheral neuropathy, n (%)	2 (8.70)	1 (2.94)	0.672	0 (0)	0 (0)	NA
Dose reduction or discontinuation due to ADR, n (%)	7 (30.43)	8 (23.53)	0.845	9 (47.37)	4 (33.33)	0.743

* *p* < 0.05; **, *p* < 0.01.

### Typical cases

When linezolid and rifampicin were used concurrently, two patients exhibited a subtherapeutic plasma concentration of linezolid. Subsequently, a significant increase in the plasma concentration of linezolid was observed in two patients after rifampicin was discontinued. Both cases are detailed below.

### Case 1

A 34-year-old man was found to have *M. tuberculosis* DNA in bronchoalveolar lavage fluid after testing in another hospital. Next-generation sequencing revealed 215 sequences of *M. tuberculosis* complex. He was admitted to the Tuberculosis Department of ACCH on 7 May 2022. He received anti-tuberculosis treatment consisting of isoniazid injection (0.3 g, once daily (Qd)), rifampicin injection (0.45 g, Qd), ethambutol tablets (0.75 g, Qd), pyrazinamide tablets (1.5 g, Qd), and moxifloxacin injection (0.4 g, Qd). However, due to limited improvement on 11 May, linezolid injection was added (0.6 g, i.v., Q12h). Nonetheless, the C_max_ of linezolid was 7.48 mg/L on 23 May, which fell below the recommended therapeutic concentration range of 12–26 mg/L. Consequently, the clinical pharmacist hypothesized that rifampicin may have contributed to the reduction in the plasma concentration of linezolid, and recommended discontinuation of rifampicin injection. On the morning of 26 May, the C_max_ of linezolid was 30.13 mg/L.

### Case 2

A 53-year-old man was transferred to the Tuberculosis Department of ACCH on 22 September 2022 due to a diagnosis of active pulmonary tuberculosis confirmed by positive results for tuberculosis DNA and T-SPOT diagnosed at another hospital. He received isoniazid injection (0.3 g, Qd), rifampicin injection (0.45 g, Qd), and ethambutol tablets (0.75 g, Qd) as anti-tuberculosis treatment, as well as meropenem injection (1.0 g) every 6 h (Q6h), ornidazole injection (0.5 g, Q8h), and penicillin sodium injection (4 million U, Q4h) to fight infection. On 26 September, linezolid injection (0.6 g, i.v., Q12h) was added to cover Gram-positive bacteria and *M. tuberculosis*. The C_min_ for linezolid was only 0.40 mg/L on 29 September. The clinical pharmacist suggested that the significantly low C_min_ for linezolid was related to co-treatment with rifampicin, and recommended discontinuation of rifampicin. Therefore, rifampicin injection was ceased on 1 October. On 4 October, the C_min_ for linezolid was 0.85 mg/L. On 8 October, the C_min_ for linezolid was 1.59 mg/L.

## Discussion

This clinical study is the first retrospective analysis of interactions between linezolid and rifampicin in Chinese patients. Patients in the linezolid + rifampicin group exhibited significantly lower plasma concentrations of linezolid (C_max_ and C_min_) compared with those in the linezolid group. The plasma concentration of linezolid of two patients increased obviously after withdrawal of rifampicin, and the plasma concentration of linezolid decreased by ∼75% when linezolid was combined with rifampicin. Hoyo *et al.* reported that in two patients, the C_min_ for linezolid during co-administration with rifampicin was reduced by >50% compared to when rifampicin was discontinued (Hoyo et al., 2012). Ashizawa et al. reported that a 79-year-old woman for whom rifampicin was added had a significant decrease in the C_min_ of linezolid (48.20%–75.50%) compared with when linezolid was administered alone (Ashizawa et al., 2016).

The distribution of plasma concentrations of linezolid exhibited variations among patients in the linezolid and linezolid + rifampicin group. Specifically, the proportion of patients with a high plasma concentration of linezolid was greater in the linezolid group compared with that in the linezolid + rifampicin group [23.81% (10/42) vs. 4.35 (2/46)]. Conversely, the proportion of patients with a lower plasma concentration of linezolid in the linezolid + rifampicin group was greater than that in the linezolid group [30.43% (14/46) vs. 23.81% (10/42)]. A 10-year retrospective study involving 1,049 patients who received linezolid (0.6 g, Q12h) and including 2,484 C_min_ points was undertaken. Results showed that 50.8% of C_min_ values were within the reference concentration range, and the prevalence of linezolid overexposure (33%) was significantly higher than linezolid underexposure (16.2%) (Pea et al., 2017). Studies have reported that linezolid overexposure was significantly associated with advanced age and creatinine clearance rate (CrCl) < 40 mL/min, and linezolid underexposure to be significantly associated with CrCl >100 mL/min (Cattaneo et al., 2016; Pea et al., 2017). There were no significant differences in age or the glomerular filtration rate among patients in our study.

The mean value and distribution range of the plasma concentration of linezolid varied between the linezolid group and linezolid + rifampicin group, which were considered to be related to the combination with rifampicin. Recent reports have indicated a tendency toward a lower C_min_ for linezolid in patients co-administered linezolid and rifampicin (Pea et al., 2010; Morata et al., 2016). A study conducted in 2012 with 45 patients demonstrated that the C_min_ (3.71 vs. 1.37 mg/L) and AUC_24 h_ (212.77 vs. 123.33 mg/L h) of patients receiving linezolid monotherapy were significantly higher than those receiving linezolid in combination with rifampicin (Pea et al., 2010). Those reports considered rifampicin to be a P-glycoprotein inducer that can accelerate the clearance and excretion of linezolid to a certain extent. Rifampicin can induce drug-metabolism enzymes, including robust expression of CYP3A4 in the liver and small intestine, as well as expression of phase-2 drug-metabolism enzymes and drug-transport proteins such as UDP-glucuronyltransferase, sulfotransferase, P-glycoprotein, multi-drug resistance protein-2, and organic anion-transporting polypeptide. Rifampicin exerts a considerable impact on the pharmacokinetics of orally administered drugs metabolized by CYP3A4 and transported by P-glycoprotein, resulting in reduced drug concentrations after metabolism by CYP3A4 and CYP2C (Niemi et al., 2003; Semvua et al., 2015). However, the mechanisms of the interaction between linezolid and rifampicin require further exploration.

Linezolid is not metabolized directly by CYP enzymes. However, the optimal conditions for the formation of linezolid metabolites are alkaline pH (9.0), suggesting involvement by an uncharacterized P450 enzyme or an alternative microsomal-mediated oxidative pathway (Wynalda et al., 2000). A study on the pharmacokinetics of linezolid in healthy people showed that co-administration of linezolid and rifampicin resulted in an earlier time for linezolid to reach the maximum concentration (T_max_): 0.24 h. In addition, prior administration of rifampicin can increase CYP3A activity in human hepatocytes, leading to a 1.3–1.6-times increase in the metabolism of linezolid (Gandelman et al., 2011). In 2018, a prospective study showed that multiple administrations of rifampicin reduced the concentration of linezolid (p.o.) at normal doses by an average of 65% (Hashimoto et al., 2018). Animal experiments revealed that multiple administrations of rifampicin resulted in reductions of 48%, 54%, and 48% in the AUC, C_max_, and oral bioavailability, respectively. However, intestinal-permeability tests conducted on rifampicin-pretreated rats and control rats indicated no disparity in the absorption and secretion of linezolid across upper, middle, and lower intestinal tissues. Those results indicate that the primary cause for the interaction between linezolid and rifampicin may be the first-pass effect exerted by the liver (Hashimoto et al., 2018). One study showed that rifampicin pretreatment of mice resulted in a reduction in the blood concentration of linezolid and decrease in AUC of ∼30%, implying that rifampicin may inhibit the absorption and accelerate the elimination of linezolid (Lampe et al., 2019).

Studies have shown that a combination of linezolid and rifampicin can reduce the prevalence of thrombocytopenia and the hemoglobin level in patients (Legout et al., 2010). Reported high-risk factors of linezolid-induced thrombocytopenia and reduction in the hemoglobin level include low platelet count, low body weight, low level of albumin, old age, longer duration of medication, renal insufficiency, and C_min_ >8 mg/L (Chen et al., 2012; Lin et al., 2022). Duration of linezolid therapy >14 days and renal insufficiency are independent high-risk factors of ADRs in the blood system (Hirano et al., 2014; Hanai et al., 2016). In the present study, there was no significant difference in the prevalence of hematological ADRs between the two groups, which may have been because of the significantly longer duration of linezolid use in the linezolid + rifampin group compared with that in the linezolid group. A report had demonstrated a significantly higher prevalence of C_min_ >10 mg/L for linezolid when linezolid was combined with P-glycoprotein inhibitors such as omeprazole, amiodarone, or amlodipine (Pea et al., 2010), but this phenomenon was not observed in our study.

The main limitation of our study was the small study cohort (especially C_min_), which limited the robustness of statistical analyses between the two groups. In a follow-up study, we will utilize a larger study cohort to confirm our findings.

## Conclusion

The plasma concentration of linezolid in the linezolid + rifampicin group was significantly lower than that in the linezolid group. In two patients, co-administration of rifampicin resulted in a 75% reduction in the plasma concentration of linezolid. However the total therapeutic efficiency and prevalence of hematologic ADRs were not significantly different in the linezolid group and linezolid + rifampin group. TDM was shown to be an important tool for evaluating the efficacy and safety of long-term treatment with linezolid combined with rifampicin in patients suffering from tuberculosis and infection. This study may improve understanding of the interactions between linezolid and rifampicin.

## Data Availability

The raw data supporting the conclusion of this article will be made available by the authors, without undue reservation.
